# Editorial: Behavioral and physiological bases of attentional biases: paradigms, participants, and stimuli

**DOI:** 10.3389/fpsyg.2015.00686

**Published:** 2015-05-22

**Authors:** Daniela M. Pfabigan, Ulrich S. Tran

**Affiliations:** ^1^Social, Cognitive and Affective Neuroscience Unit, Department of Basic Psychological Research and Research Methods, Faculty of Psychology, University of ViennaVienna, Austria; ^2^Department of Basic Psychological Research and Research Methods, Faculty of Psychology, University of ViennaVienna, Austria

**Keywords:** attentional bias, bias indices, attentional ERPs, anxiety, depression, dot-probe task

Processes of selective allocation of visual attention play a prominent role for survival, but also for development and maintenance of clinically relevant symptoms such as in anxiety or depression. Previous research provided evidence for increased attentional orienting and preoccupation with biologically relevant and mood-congruent stimuli, indicating tendencies of attentional biases. For instance, in anxiety, the visual-attentional system may be overly sensitive toward threat- and avoidant of reward cues.

The research articles appearing in the E-book *Behavioral and physiological bases of attentional biases: paradigms, participants, and stimuli* cover these topics and give a comprehensive overview on current directions and challenges in attentional bias research. Our driving motivation was to critically evaluate parameters that may directly or indirectly influence attentional biases and may thus be important for our understanding of attentional biases.

Our first aim was to demonstrate the variety of experimental paradigms and outcome measures used. So far, the *dot-probe task* (MacLeod et al., 1986) was the gold standard in attentional bias research. This was also reflected in the contributions to this research topic. Relying solely on behavioral measures such as response accuracy, response times, and bias indices, Hakamata et al. (2014) applied a dot-probe task whereas Sagliano et al. (2014) and Wittekind et al. (2015) applied (modified) versions of the Posner task (Posner, 1980), a cueing paradigm related to the dot-probe task. In contrast, Isomura et al. (2014) applied an innovative combination of two tasks—a search-recognition and a face-in-the-crowd task. Relying on physiological measures, Valuch et al. (2015) applied a gap-saccade and a dot-probe task while measuring saccadic reaction times. Focusing on the exact time course of neuronal activation, four studies investigated attentional biases using electroencephalography and measured attention-related event-related potentials (ERPs) in response to emotional and neutral stimuli. Sass et al. (2014) and Fisher et al. (2014) applied an emotion word Stroop task while Pfabigan et al. (2014) and Kappenman et al. (2014) administered versions of the dot-probe task. These studies investigated several attention-related ERPs time-locked to crucial events during the paradigms—pointing toward the huge diversity in measures used to assess attentional biases.

Our second aim was to demonstrate the variety of populations and stimuli, which may show, or elicit, differing attentional biases. Wittekind et al. (2015) examined individuals who had experienced displacement during World War II and their adult offspring. There was no evidence of attentional biases among formerly displaced individuals suffering from post-traumatic stress disorder (PTSD), and no evidence of transgenerational transmission of attentional biases in PTSD, utilizing pictorial stimuli. Only when utilizing word stimuli, evidence of trauma-related attentional biases among participants with PTSD could be replicated, highlighting that attentional biases may sometimes depend on the type of stimuli used. Fisher et al. (2014) examined subclinical samples, investigating the moderating effect of suspiciousness on attentional biases in anxiety and depression. Utilizing word stimuli, there was evidence of overlapping processes for suspiciousness and anxious apprehension, but not for suspiciousness and depression. Isomura et al. (2014) examined children with autism spectrum disorders (ASD), utilizing facial stimuli. Children with ASD showed quicker detection of angry faces than typically developed children, relying more on the extraction of local than configural features during face processing. Valuch et al. (2015) investigated the effects of perceived attractiveness in facial stimuli among healthy participants. Attractive faces captured attention more effectively than less attractive faces, and men showed a stronger bias toward attractive opposite-sex faces than women. Utilizing pictorial stimuli, Kappenman et al. (2014) examined healthy participants, whereas Sagliano et al. (2014) contrasted high and low anxious subclinical participants. Sass et al. (2014) examined subclinical samples, utilizing word stimuli, and found evidence of differential attentional biases among men and women and effects of co-occurring anxiety in attentional bias in depression. Sex differences were also evident examining a subclinical sample utilizing facial stimuli (Pfabigan et al., 2014).

Investigating the impact of participant sex on attentional biases, Pfabigan et al. (2014) applied a modified version of the dot-probe task which included also neutral-neutral stimulus pairs (Koster et al., 2004). These trials allowed a distinction of attentional allocation and disengagement processes. Importantly, this approach can also be applied to physiological data by calculating so-called ERP difference waves (i.e., subtracting neuronal activity evoked by neutral stimuli from the neuronal activity evoked by emotional ones), which allows the disentanglement of attentional processes also on the neuronal level. Despite limitations of the dot-probe task, this approach might be considerably useful in future attentional bias research.

Kappenman et al. (2014) investigated the reliability of reaction time measures, bias indices, and the N2pc ERP component in a standard picture-based dot-probe task. The authors reported poor reliability of behavioral measures, but moderate reliability of the N2pc component. They emphasize the need for experimental paradigms that are better suited for the assessment of attentional biases and they advocate including physiological measures to gain more reliable insight into the underlying processes.

Challenges in attentional bias research become quite clear in the course of the articles in this E-book. There is no common agreement as to whether particular stimuli or experimental set-ups are more reliable than others. For example, only a few studies addressed the topic of different presentation durations and their impact on attentional bias measures so far (e.g., Koster et al., 2007; Mingtian et al., 2011). Moreover, the dependent variables used to assess attentional biases vary considerably, in particular in physiological studies in which ERPs in varying time windows and electrode locations are reported. This limits the comparability of studies and does not allow generalizable conclusions.

Nevertheless, the current research topic also points toward future directions of attentional bias research. In particular, Kappenman et al. (2014) emphasize the need for task development to assess attentional biases in a more reliable way. The study by Isomura et al. (2014) should be considered pioneering in this regard. Moreover, using statistical methods that account for random variance due to stimulus variation (Judd et al., 2012) or applying difference measures also in physiological attentional bias research (Pfabigan et al., 2014) might be promising for research in this field.

## Conflict of interest statement

The authors declare that the research was conducted in the absence of any commercial or financial relationships that could be construed as a potential conflict of interest.
